# Feasibility of free-breathing quantitative myocardial perfusion using multi-echo Dixon magnetic resonance imaging

**DOI:** 10.1038/s41598-020-69747-9

**Published:** 2020-07-29

**Authors:** Cian M. Scannell, Teresa Correia, Adriana D. M. Villa, Torben Schneider, Jack Lee, Marcel Breeuwer, Amedeo Chiribiri, Markus Henningsson

**Affiliations:** 1 School of Biomedical Engineering and Imaging Sciences, King’s College London, London, UK; 2 Philips Healthcare, Guildford, UK; 3 Philips Healthcare, Best, The Netherlands; 4 Department of Biomedical Engineering, Medical Image Analysis Group, Eindhoven University of Technology, Eindhoven, The Netherlands; 5 Division of Cardiovascular Medicine, Department of Medical and Health Sciences, Linköping University, Linköping, Sweden; 6 Center for Medical Image Science and Visualization (CMIV), Linköping University, Linköping, Sweden

**Keywords:** Cardiology, Cardiovascular diseases, Magnetic resonance imaging

## Abstract

Dynamic contrast-enhanced quantitative first-pass perfusion using magnetic resonance imaging enables non-invasive objective assessment of myocardial ischemia without ionizing radiation. However, quantification of perfusion is challenging due to the non-linearity between the magnetic resonance signal intensity and contrast agent concentration. Furthermore, respiratory motion during data acquisition precludes quantification of perfusion. While motion correction techniques have been proposed, they have been hampered by the challenge of accounting for dramatic contrast changes during the bolus and long execution times. In this work we investigate the use of a novel free-breathing multi-echo Dixon technique for quantitative myocardial perfusion. The Dixon fat images, unaffected by the dynamic contrast-enhancement, are used to efficiently estimate rigid-body respiratory motion and the computed transformations are applied to the corresponding diagnostic water images. This is followed by a second non-linear correction step using the Dixon water images to remove residual motion. The proposed Dixon motion correction technique was compared to the state-of-the-art technique (spatiotemporal based registration). We demonstrate that the proposed method performs comparably to the state-of-the-art but is significantly faster to execute. Furthermore, the proposed technique can be used to correct for the decay of signal due to T2* effects to improve quantification and additionally, yields fat-free diagnostic images.

## Introduction

Myocardial perfusion can be assessed using dynamic MRI during first-pass of a contrast agent^1^. While the images are routinely reviewed visually in the clinic, quantification is desirable as it is user-independent^2^. Quantification of myocardial perfusion can be challenging mainly due to the non-linearity between the signal intensity and contrast agent concentration at concentrations necessary to observe potential perfusion abnormalities within the myocardium^3^. Furthermore, respiratory motion makes the quantification of perfusion difficult, and may even preclude it.

Although most commonly used to mitigate respiratory motion, breath-holding for at least 40 s (to cover the full first pass of the contrast bolus) is challenging for many patients, particularly under stress conditions. Moreover, long breath-holds can lead to changes in heart rate, resulting in images being acquired at slightly different cardiac phases thus introducing cardiac motion. Conversely, if patients are allowed to breathe normally, respiratory motion tends to be more regular and shallower when compared to the large gasps that may occur when the patient can no longer hold their breath. Therefore, acquisitions in free-breathing are easier to correct for respiratory motion using image registration compared to breath-holding with intermittent breathing. The problem of registering myocardial perfusion MR images is, however, challenging due to the rapid change in signal intensity during the contrast bolus transit. The dynamic contrast-enhancement invalidates the assumptions of constant intensity cost functions that are optimised in such schemes and as a result, intensity-based registration techniques may not be readily applied.

Several image registration methods have been proposed to correct for respiratory motion between time frames in myocardial perfusion MRI^4–7^. However, to date, no consensus exists as to which method should be use clinically. Some approaches correct for rigid motion only to mitigate for the most severe breathing artefacts, since a substantial part of the motion is due to respiratory motion in the head-foot direction^7–11^. Rigid registration methods are computationally efficient, consistent and robust to noise. However, these do not capture more complex non-rigid deformations and hence, images are not precisely aligned. Non-rigid registration methods provide a better alignment of the heart during breathing, but are susceptible to noise and are computationally expensive, and thus, can be impractical for use in a clinical setting^12–17^. In addition, non-rigid methods can cause blurring and non-physiological geometric deformation of the heart, hampering accurate myocardial perfusion quantification. However, non-rigid registration performs better when correcting for small misalignments. Therefore, adding an initial rigid registration step results in a more effective registration than a purely non-rigid transformation in the presence of large motion^18^. Tracking of respiratory motion may be facilitated by separating water and fat signal using multi-echo Dixon (mDixon) imaging, which has previously been implemented for renal perfusion MRI^19^. Fat images may then be used to estimate rigid respiratory motion, as there is no local signal intensity change, and this facilitates the use of simple intensity-based registration methods. The transformations computed to correct the fat images can subsequently be applied to correct the corresponding diagnostic water images.

The mDixon MRI acquisitions also provide fat-free diagnostic images, thereby avoiding the need for a fat-suppression pulse to null the signal from (epicardial) fat. In myocardial perfusion MRI fat suppression is important^2,20^ to minimise potential partial volume effects at the myocardial-epicardial border and to improve the accuracy of myocardial blood flow quantification. Moreover, accurate myocardial perfusion quantification depends on the accurate measurement of the arterial input function (AIF). However, the true AIF is affected by the non-linear response of the saturation recovery signal and T2*-related losses at high contrast agent concentrations in the blood pool. To ameliorate these effects, the dual-bolus^21^ and dual-sequence^2^ imaging strategies have been proposed. The dual-bolus method uses a low dose bolus to measure the AIF and a high dose bolus for myocardial analysis. In the dual sequence method, a low-resolution AIF image is acquired using very short echo times to minimise T2*-related signal loss. In addition, a dual-echo acquisition has been used to further correct the effect of T2* losses on the AIF^22^. However, these techniques require multiple injections and/or acquisitions.

In this work, we investigate the merits of quantitative perfusion with mDixon for respiratory motion correction, fat suppression and T2* correction of the AIF. For the respiratory motion correction, a two-step approach is proposed, whereby first the fat images are used to estimate the bulk rigid-body motion of the heart, with the transformation then being applied to the diagnostic water images. This is followed by a second step to minimise residual motion with a non-rigid correction using only the water images. The new technique is evaluated in 14 patients with normal myocardial perfusion during rest.

## Methods

### MRI acquisition

**Figure 1:**
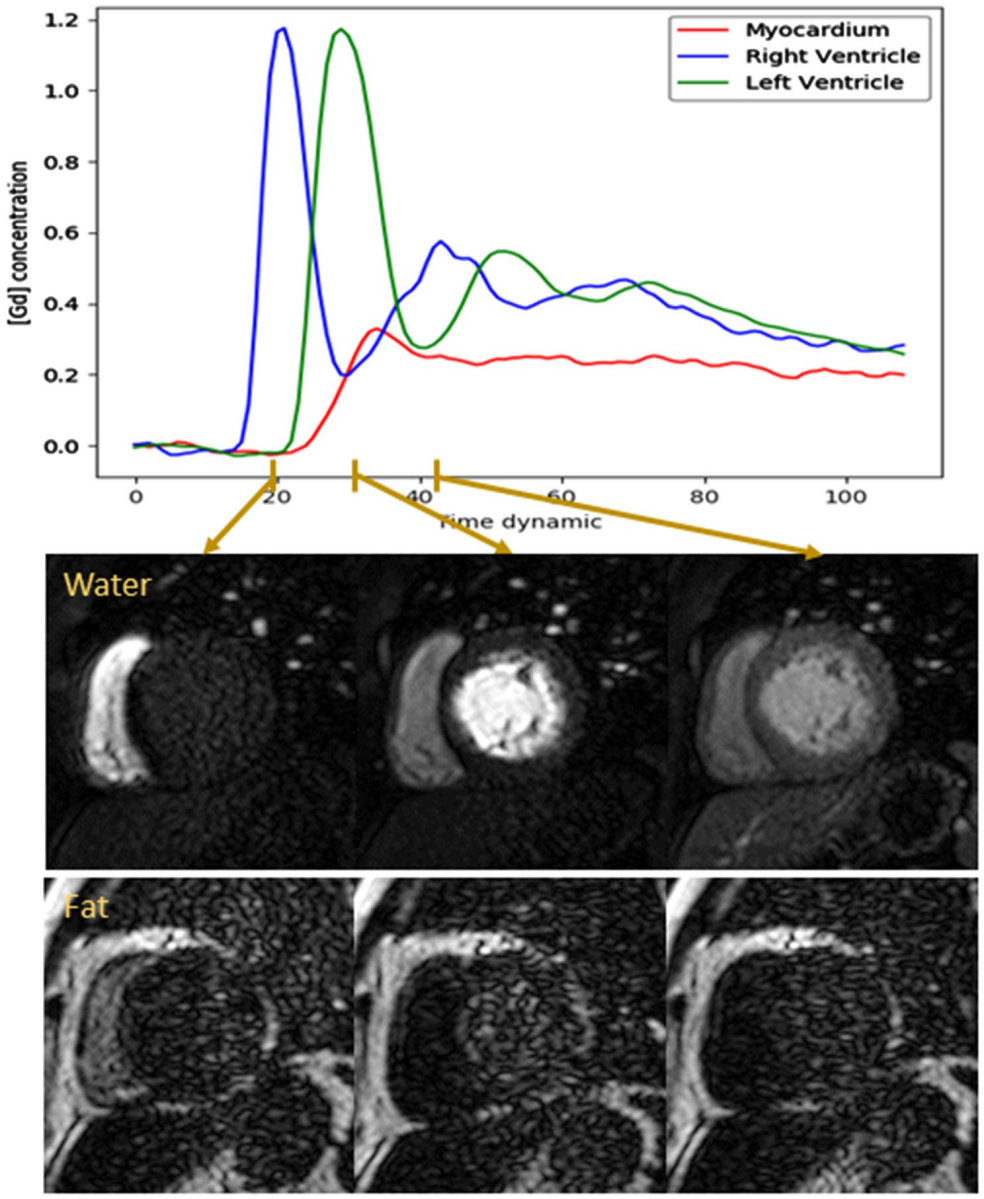
The fat–water separation of three example frames. The arrows indicate the time points of the main bolus of contrast agent that the dynamics are taken from (peak enhancement in the right ventricle (divided by 3), peak enhancement in the left ventricle (divided by 3) and peak myocardial enhancement). The fat images are free of the dynamic contrast-enhancement that precludes image registration, while retaining enough structural information to allow rigid correction of the respiratory motion.

The proposed acquisition consisted of a spoiled gradient echo readout with three echoes per excitation pulse to enable T2* estimation and water-fat separation using Dixon reconstruction^23^. All MRI acquisitions were performed on a 3.0 T Achieva scanner (Philips Healthcare, Best, The Netherlands) using a 32-channel cardiac coil. Imaging parameters included spatial resolution: 2.5 × 2.5 × 10 mm^3^, flip angle: 14°, FOV: 360 × 310 mm, TR/TE1/ΔTE: 3.6/1.0/0.9 ms, SENSE: 2, partial Fourier: 0.63, profile order: linear, acquisition window: 127 ms, saturation delay: 75 ms, three slices (base, mid, and apical) per heart-beat. A WET pulse was used for signal saturation^24^. Example water-fat separated perfusion images are shown in Fig. 1 demonstrating constant fat signal despite dramatic changes in water signal due to contrast agent bolus passing. Example videos of corresponding water and fat image series are provided in the supplementary material as Supplementary Videos S1–S4.

### Respiratory motion correction

**Figure 2:**
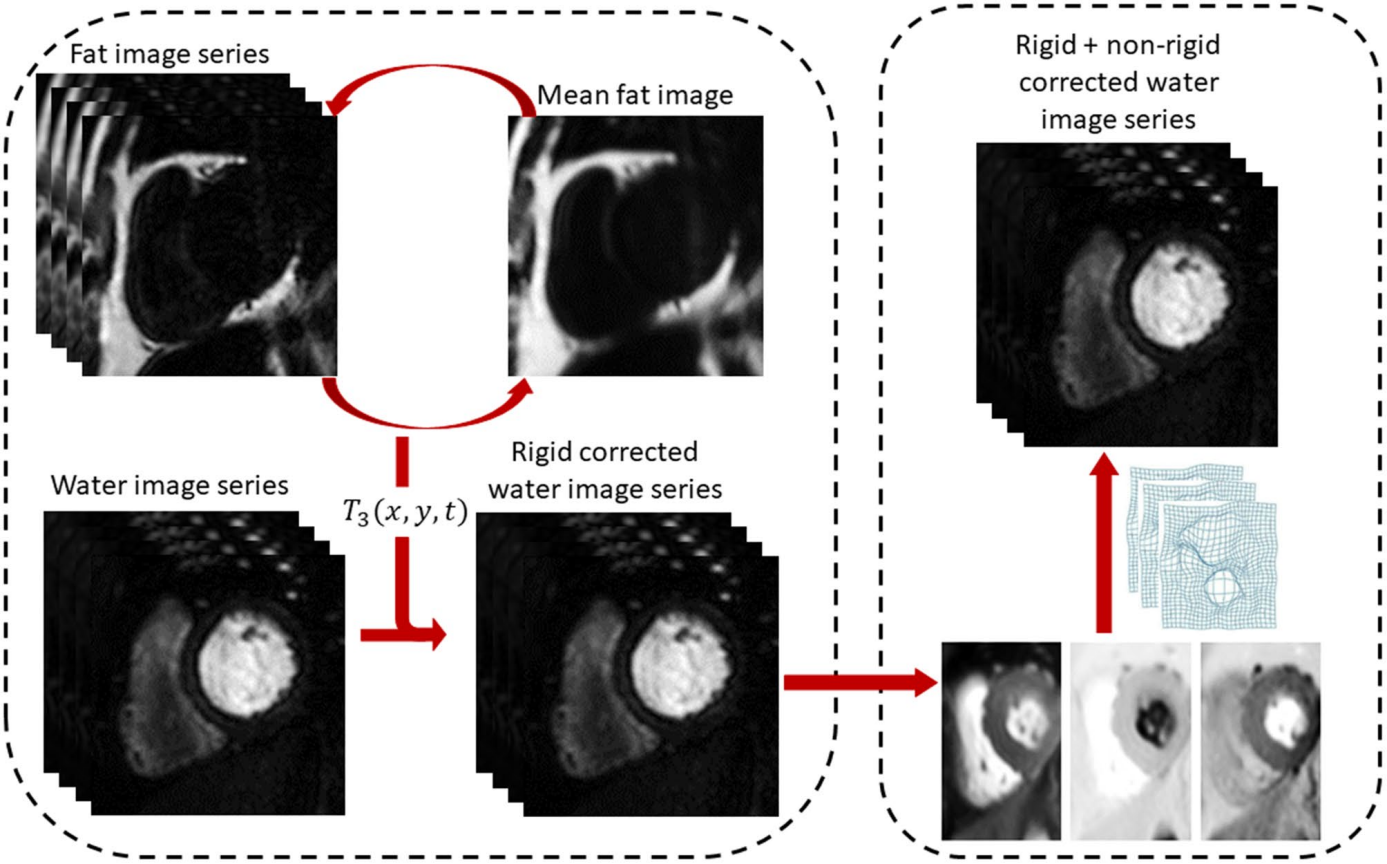
A flow chart which describes the proposed two-step motion correction scheme. The fat images obtained from the multi-echo Dixon reconstruction are used to correct for the rigid body bulk motion. This is done in an iterative fashion. For three iterations the fat images are registered to the mean fat image, which updates on each iteration, resulting in transformations ${T}_{1}\left(t\right),{ T}_{2}(t)$ and ${T}_{3}(t)$ for each time dynamic $t$. The composition of these three transformations ${T}_{1-3}(t)$ is then applied to the diagnostic water images. The water image which have been rigidly corrected are then refined through non-rigid registration to a synthetic PCA-based image series.

The proposed motion correction approach, which is conducted in two stages, is illustrated in Fig. 2. First, water and fat images were generated using mDixon reconstruction^23^. A rectangular region of interest surrounding the left ventricle was automatically computed using our previously described deep learning-based processing pipeline^25^ and the fat images were then registered in an iterative manner using a rigid (translation and rotation) transformation to optimise the mean squared error (MSE) cost function. The reference frame was taken to be the mean frame of the image series. Each image in the series was registered to the reference image and this process was repeated for three iterations. The computed transformations were then applied to the corresponding water image series in order to correct for the rigid body respiratory motion.

The second step involved using a non-linear registration algorithm and was applied to the water image series to minimise residual respiratory motion. This stage was based on the observation that after the rigid body motion correction the remaining motion consists only of random fine misalignments. Hence, this motion appears only in the later components of a principal component analysis (PCA) decomposition^26,27^. This is because a principal component represents the feature which accounts for most of the variance in the data set that has not been accounted for by a previous principal component. Since the residual motion after rigid body correction will be small, it will not appear in the early principal components. It is thus possible to create a motionless synthetic reference image series using only the early principal components that has the same dynamic contrast-enhancement as the original series. In this work, the number of principal components used was chosen, empirically, to be three. This is a natural choice due to the structure of the data as first three principal components represent the largest modes of variation in the dataset. In the case of a myocardial perfusion image series, these are the enhancement of the left ventricle, the enhancement of the right ventricle and the perfusion of the myocardium. It is required to include all three of these components in order to retain enough information so that the synthetically generated image series has the same dynamic contrast-enhancement pattern as the original image series. This step would not be feasible without the earlier rigid body motion correction as in this case the motion would be significant enough to appear in these early principal components. Each image in the water image series is then registered to the corresponding PCA-based synthetic image using free-form deformations^28^, optimising the residual complexity cost function^29^. The motion correction was implemented in Matlab (The MathWorks, Natick, MA, USA) using software developed in-house with the Medical Image Registration Toolbox for Matlab^30^. Videos demonstrating the image series at different stages of the motion correction pipeline (water/fat images before motion correction, water/fat images after rigid motion correction and water images after both rigid and non-rigid motion correction) are shown in the supplementary material as Supplementary Videos S3–S7.

### In vivo experiments

The proposed perfusion technique was performed in fourteen patients referred to our CMR centre for function and viability assessment. No perfusion defects were expected. The study was approved by the North of Scotland Research Ethics Committee, United Kingdom (ethics approval number 15/NS/0030). All patients gave written informed consent prior to participation and all experiments were carried out in accordance with relevant guidelines and regulations. Perfusion scans were performed at rest over 1 min and 20 s during free-breathing with a dual-bolus contrast injection of 0.05 mmol/kg^21^. The pre-bolus was diluted to 10% concentration of the main bolus.

Quantitative MBF maps were computed with no motion correction (no MoCo), after motion correction using the mDixon fat images (Dixon MoCo) and after motion correction with a state-of-the-art approach^12,31^, using the water images. This algorithm iteratively registers the image series to a spatio-temporal denoised reference image series with the aim of progressively removing the motion (S-T denoising). The open-source implementation of this approach was used^12^.

Due to the absence of ischaemia and scar in the patients and the fact the perfusion imaging was performed under resting conditions, no perfusion defects should be observed, resulting in uniform quantitative perfusion maps. This is not the case in the presence of motion as this introduces artefacts in the time-intensity curves. In order to assess the efficacy of the motion correction, the temporal smoothness of the time-intensity curves was analysed. To this end, the second derivatives of the pixel-wise time-intensity curves were computed in the myocardium. The standard deviation (SD) of this is then computed for each curve and the mean value is computed over all curves from an individual slice, as previously suggested^32^. In the absence of motion the intensity changes in the curves should be smooth and thus lower values of this metric are associated with lower variations in the curves and more effective motion correction.

### Perfusion quantification

The perfusion images generated with the different methods were processed automatically using our deep learning processing pipeline^25^ and MBF is quantified using the dual-bolus AIF. Pixel-wise time signal intensity curves were then extracted from the myocardial mask. Signal intensity curves were subsequently split into the time intervals corresponding to the pre bolus injection and the main bolus injection for quantification. Quantitative myocardial blood flow (MBF) was estimated on a pixel-wise level by fitting the observed AIF and myocardial tissue curves to a two-compartment exchange model^33^, as proposed for quantitative myocardial perfusion analysis by Jerosch-Herold^1^ and as described by the pair of coupled ordinary differential equations (ODEs):

1 $${v}_{p}\frac{d{C}_{p}}{dt}={F}_{p}\left(\frac{{C}_{aif}\left(t\right)}{1-Hct}-{C}_{p}\left(t\right)\right)+PS \left({C}_{e}\left(t\right)-{C}_{p}\left(t\right)\right)$$

2 $${v}_{e}\frac{d{C}_{e}}{dt}=PS ({C}_{p}\left(t\right)-{C}_{e}\left(t\right))$$

In Eqs. (1) and (2), ${C}_{p}(t)$ and ${C}_{e}(t)$ are the concentration of contrast agent in the plasma and interstitial space at time $t$, respectively (in units of M). ${F}_{b}={F}_{p}/(1-Hct)$ is the myocardial blood flow (mL/min/g), ${v}_{p}$ is the fractional plasma volume (dimensionless), ${v}_{e}$ is the fractional interstitial volume (dimensionless) and $PS$ is the permeability-surface area product (mL/min/g). Hct is the haematocrit level and was taken to be 0.42^34^. The kinetic parameters were estimated from the observed data using hierarchical Bayesian inference^35^.In order to facilitate the conversion of signal intensities to concentration of gadolinium ([Gd]), a linear relationship was assumed, although this does not hold in regions of high concentrations.

### T2* correction

Typically, the AIF is affected by high concentrations of Gd, partially due to the associated T2*-related signal loss. Though the dual-bolus approach is used in this work to quantify MBF in order to negate the effects of both T1 and T2*-related signal loss, it is shown that the echo images can be used to correct the T2* effects in the main bolus. This could be used in conjunction with a dual-sequence approach in the future to account for both T1 and T2* signal loss.

**Figure 3:**
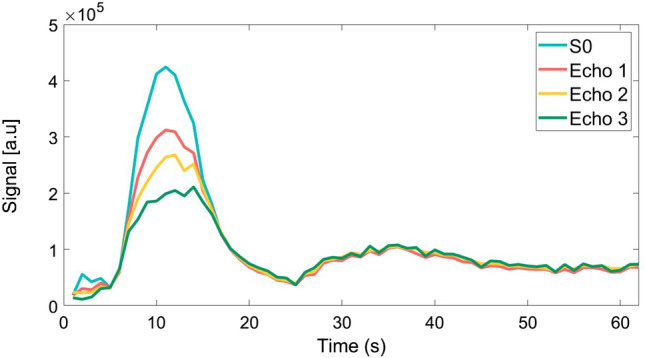
The (main-bolus) arterial input functions (AIFs) after Dixon MoCo for the three echo images with the T2* corrected AIF obtained through the fitting of the three echo AIFs to Eq. (3).

The time-varying T2* was estimated in the left-ventricular (LV) blood pool by fitting the mean signal magnitude (*S*) from the three echo images to the equation:

3 $$S(t)={M}_{0}{(t)e}^{-\frac{TE}{{T2}^{*}(t)}}$$

for each time point, where *TE* is the echo time and *M*_*0*_*(t)* is the signal at *TE* = 0 which can be considered as the T2* corrected signal. The effect of T2* correction on the AIF estimation was investigated by performing T2* correction on the AIF and comparing the peak bolus signal relative to the post-peak baseline, calculated as a percentage. This was compared to the AIF without T2* correction. The process of the T2* correction, including the three echo AIFs and the T2* corrected signal, can be visualised (for the main-bolus) in Fig. 3.

### Statistical analysis

The distribution of quantitative values computed with no MoCo were compared to those computed with Dixon MoCo and S-T denoising using the Mann–Whitney U test. This non-parametric test was chosen to avoid assumptions on the distribution of the data. A p-value cut-off level of 0.05 was chosen to indicate statistical significance.

## Results

### Motion correction

**Figure 4:**
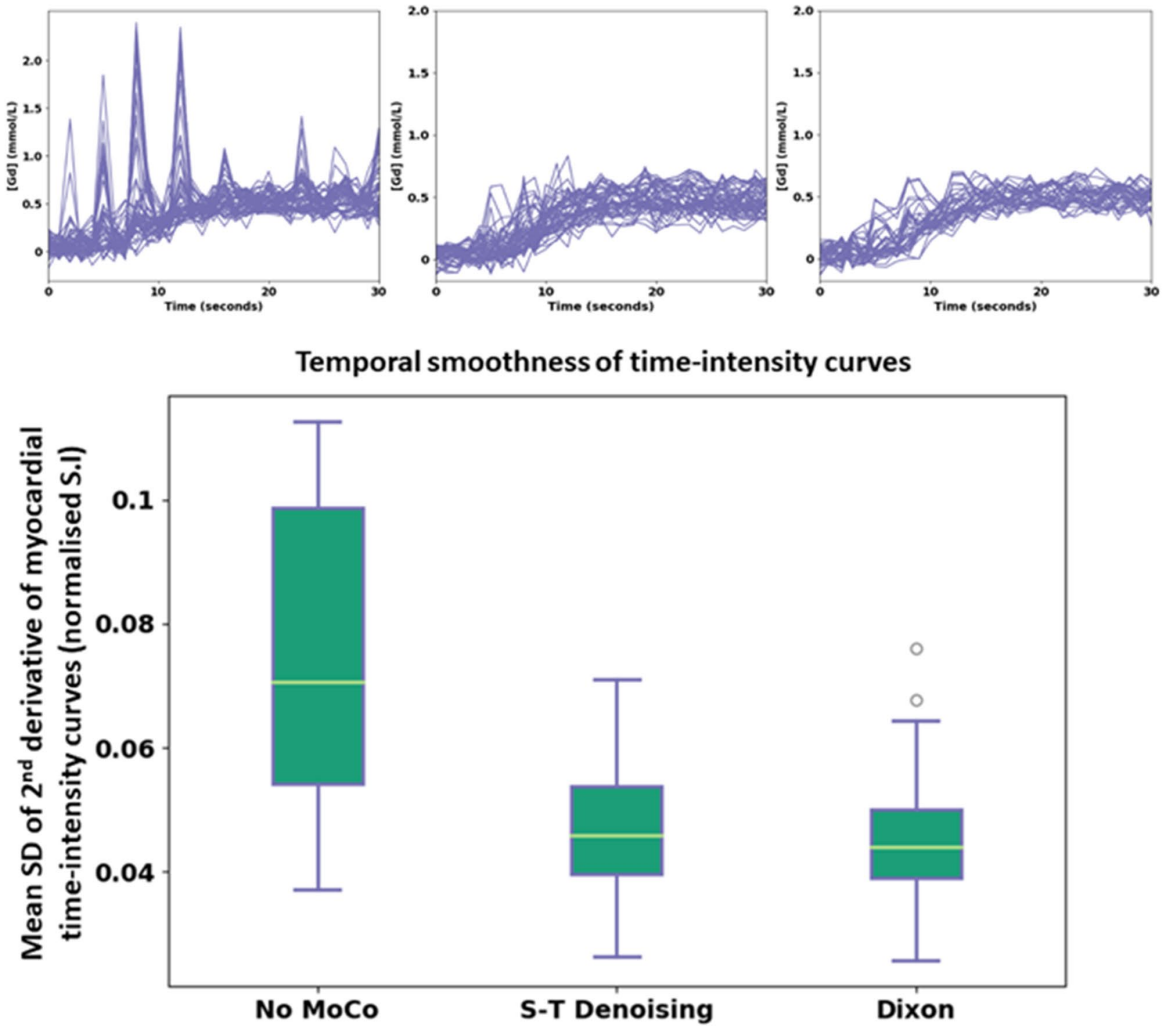
The boxplots distributions of the temporal smoothness metric for the no MoCo, S-T denoising and Dixon MoCo images respectively. As expected, the S-T denoising and Dixon MoCo images are significantly smoother than the images without motion correction. The Dixon MoCo images are seen to be marginally smoother than the S-T denoising images. The top row shows the time-intensity curves from an example image series, in the same order.

**Figure 5:**
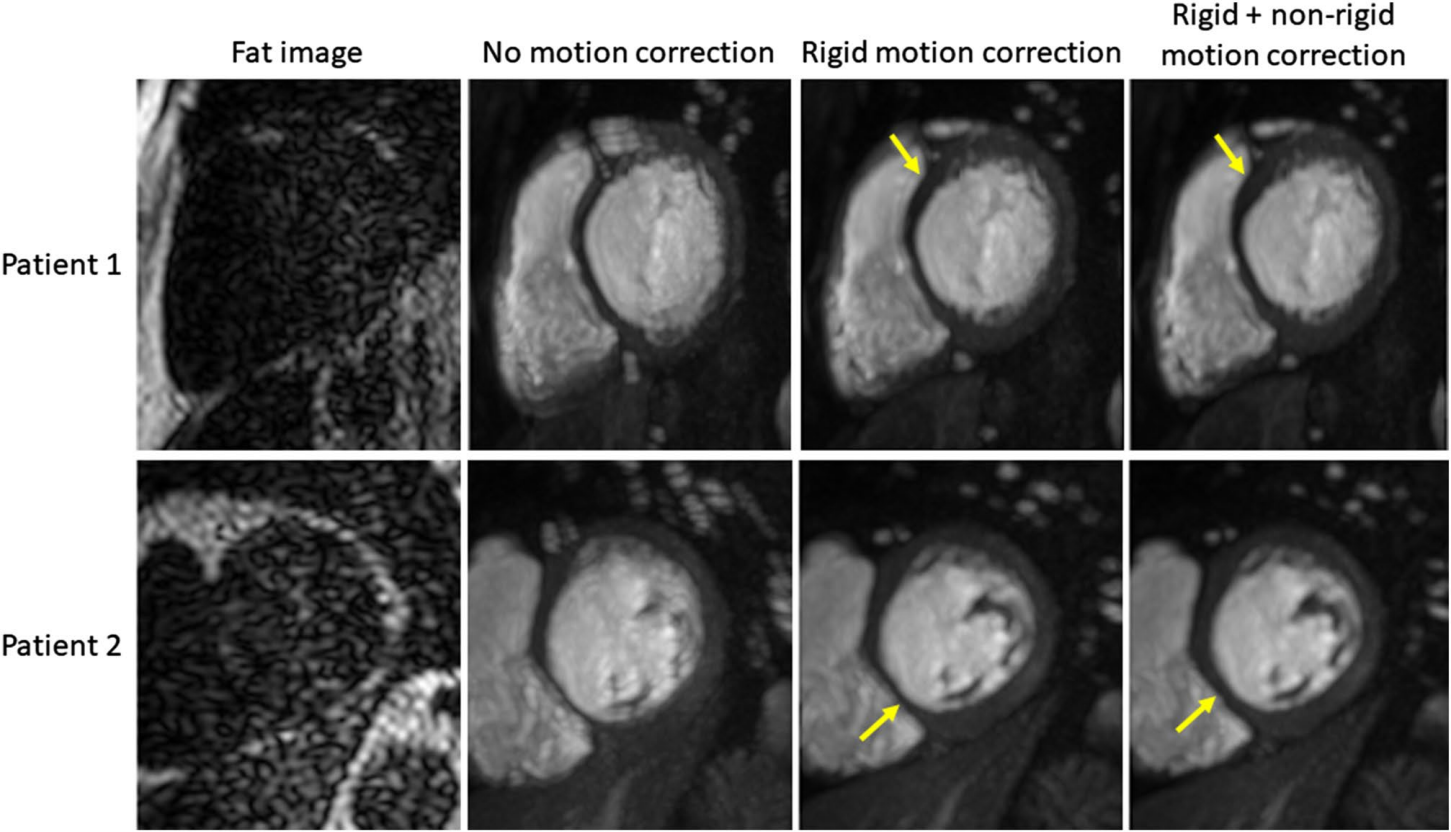
The temporal maximum intensity projection of the image series of two patients. The images shown are the basal slice from two different patients. The first column shows the corresponding fat image that was used for the rigid correction. The increased sharpness and clearly defined features that are evident after motion correction indicate a lack of motion. The arrows indicate regions where the fat images are lacking structural information and hence the second, non-rigid stage of the correction is required to accurate delineate the anatomy.

The mean (± SD) temporal smoothness values for the three motion correction states (no MoCo, S-T denoising and Dixon MoCo) were 0.076 (± 0.02), 0.047 (0.01) and 0.045 (0.01) (normalised signal intensity units), respectively. The distributions of these values are shown for the three states in Fig. 4. Both S-T denoising and Dixon MoCo yielded significantly smoother myocardial time-intensity curves than no MoCo (both p < 0.01). S-T denoising and Dixon MoCo do not differ significantly (p = 0.17). The absence of motion artefacts in the time-intensity curves is shown as increased sharpness in the temporal maximum intensity projection (tMIP) images and this is visualised in Fig. 5, before and after Dixon MoCo. The mean registration time per slice (± SD) for the S-T denoising method was 171.9 (± 44.6) seconds while the proposed Dixon MoCo method took 104.6 (± 19.7) seconds.

**Figure 6:**
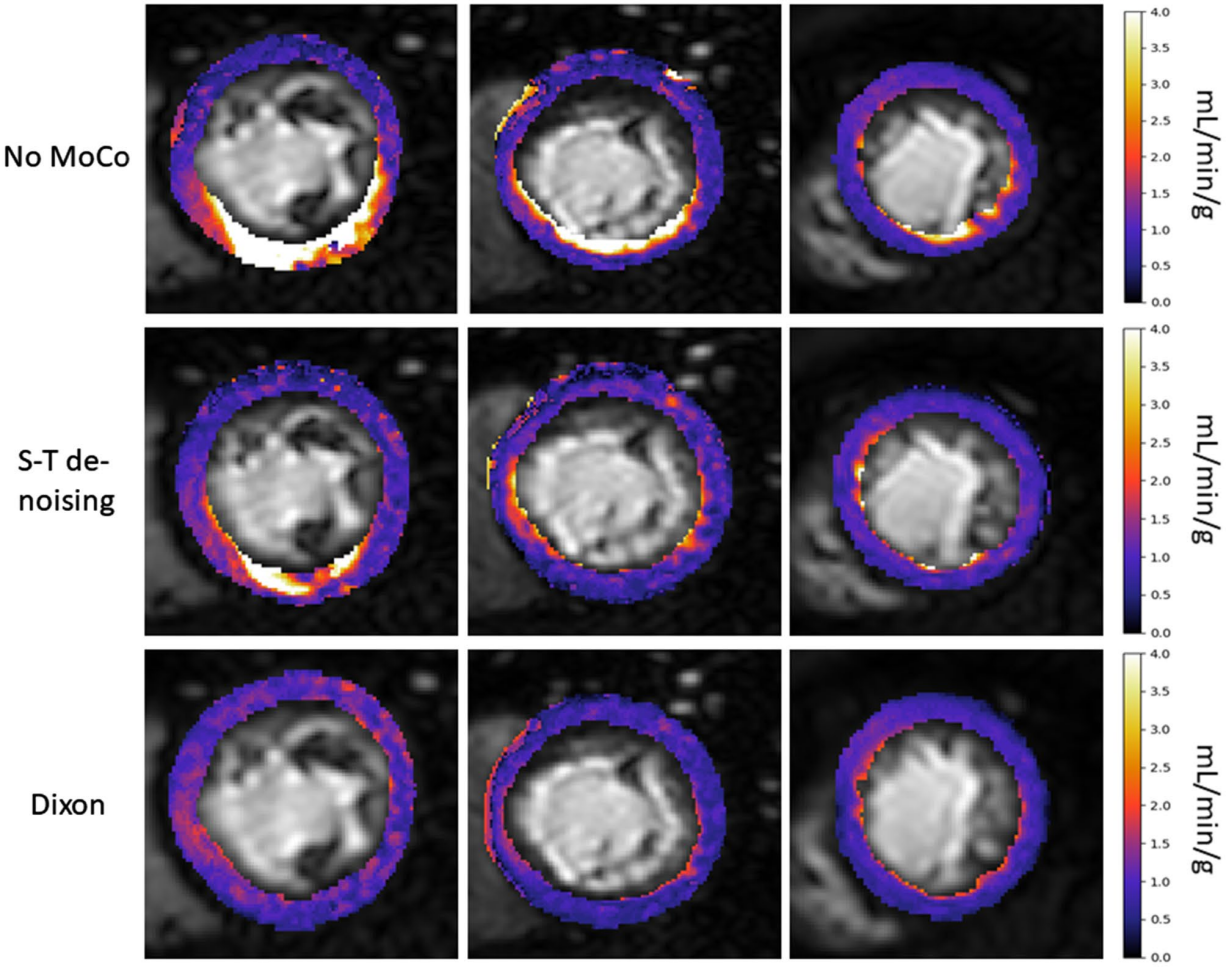
Example quantitative MBF maps for one image slice from three representative patients (columns) are shown for the three motion correction states (no MoCo, S-T denoising and Dixon MoCo, respectively) (rows). The presence of motion in the images without motion correction (no MoCo) manifests itself as spurious areas of large MBF values. This is eradicated to a certain extent in the S-T denoising images and almost completely in the Dixon MoCo images. Further MBF maps are shown in the supplementary material Fig. S1.

**Figure 7:**
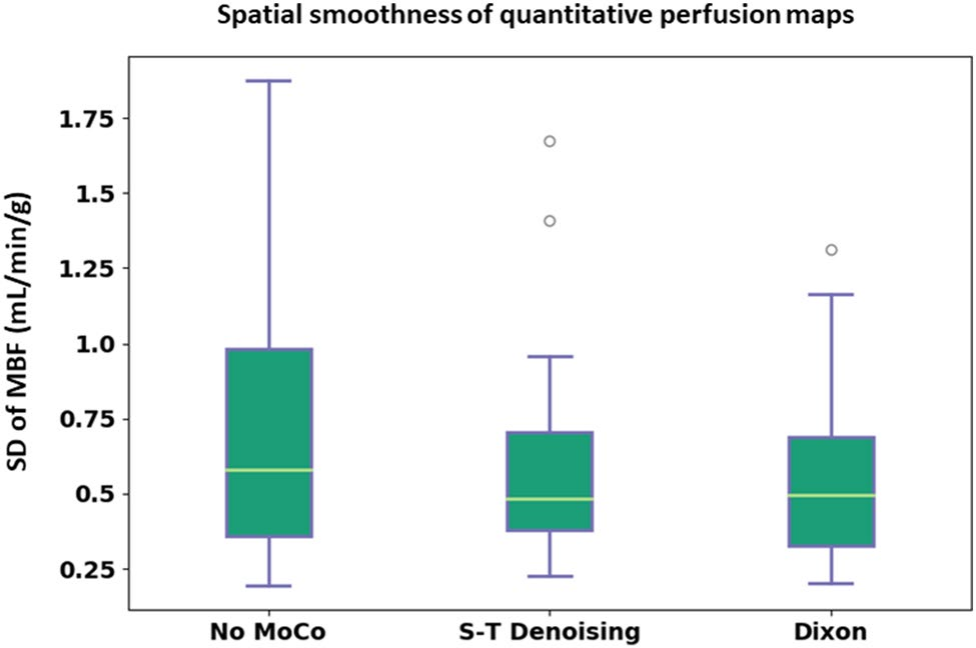
The distribution of the MBF spatial uniformity metric for the three motion correction states. The quantitative MBF maps become more uniform after motion correction due to the eradication of the motion-related artefacts in the myocardial time-intensity curves. Similar performance is reported for the S-T denoising and Dixon MoCo images with both being significantly more uniform that then no MoCo images.

**Figure 8:**
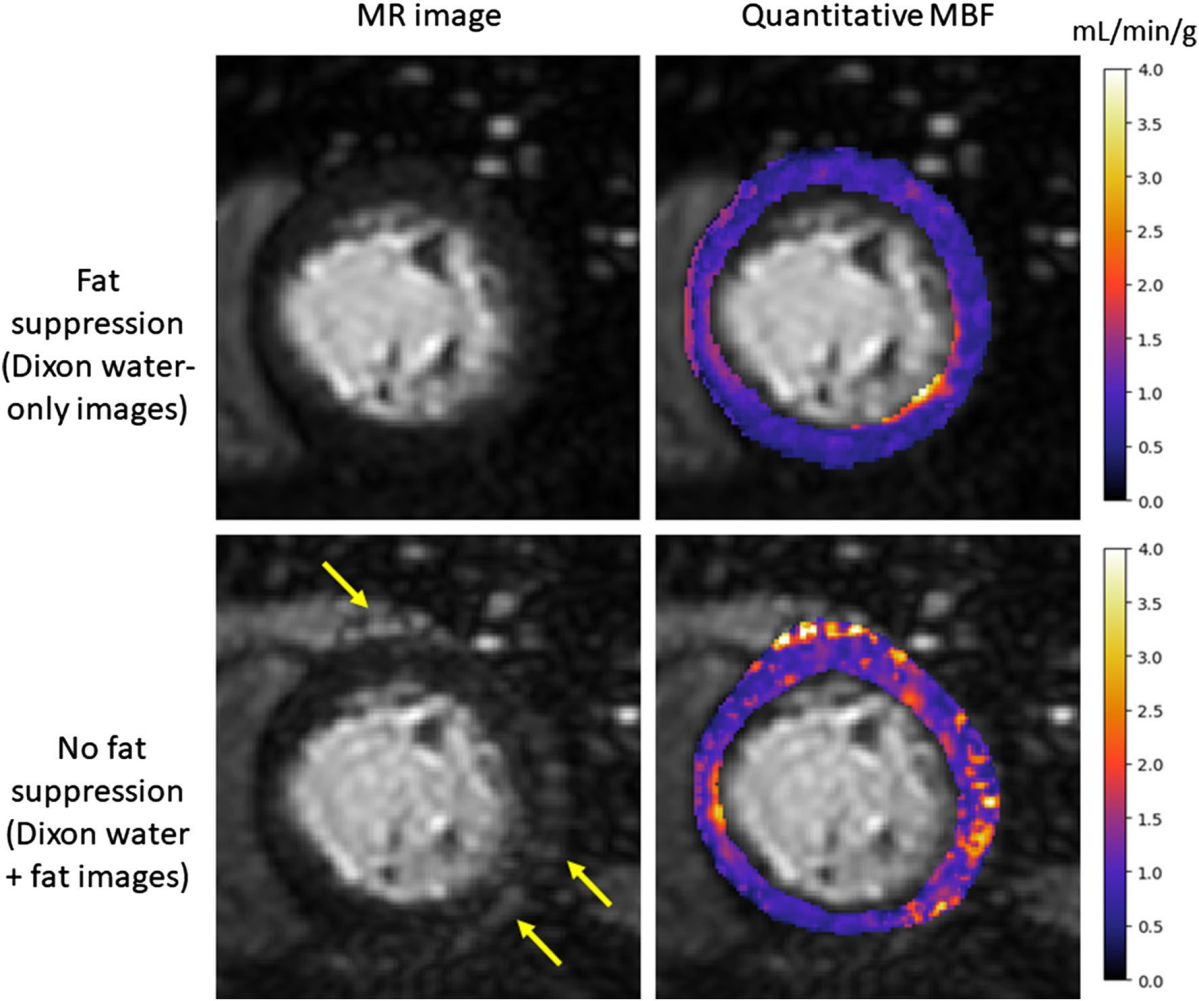
The MR images and corresponding quantitative MBF maps for a representative patient computed with both the water-only and the water and fat images. The benefit of the fat suppression is evident in the water and fat images. The fat, which appears as areas of high signal intensity (yellow arrows), leads to corresponding areas of spurious MBF values that are not present on the water-only images.

Example quantitative MBF maps are shown for slices from three patients for no MoCo, S-T denoising and Dixon MoCo images in Fig. 6. The effect of motion is visible in the spurious areas of large MBF values, particularly in the no MoCo images. This evidences the increased non-uniformity (standard deviation) of MBF maps with the presence of motion. The equivalent mean (± SD) standard deviation of the quantitative MBF maps were 0.82 (± 0.43), 0.66 (± 0.3) and 0.66 (± 0.28) mL/min/g for the no MoCo, S-T denoising and Dixon MoCo, respectively. The distributions of these values are visualised in Fig. 7. The MBF maps for the S-T denoising and Dixon MoCo images were statistically more uniform than the corresponding no MoCo maps (p = 0.046 and p = 0.044, respectively). The S-T denoising and Dixon MoCo maps were not significantly different (p = 0.44). Figure 8 shows MBF maps calculated with the water images as well as maps generated with water plus fat images. The latter image approximates the scenario where no fat suppression is used and the improved MBF maps obtained with the water only images in Fig. 8 demonstrate the value of fat suppression for MBF quantitation.

Example videos of image series before and after motion correction are provided in the supplementary material as Supplementary Videos S8 to S13.

### *T*_2_* correction

The correction for the signal loss caused by the *T*_2_* decay at high concentrations of contrast agent was achieved by fitting the three echo images to the exponential decay model given by Eq. (3) to solve for the original signal amplitude. The mean (SD) increase in peak signal intensity was 6.2% (± 5.95%) compared to the uncorrected arterial input function with a maximum increase of 20.13%.

## Discussion

In this study a multi-echo Dixon acquisition was used for first-pass myocardial perfusion, which allowed the separation of the water and fat signals. The separation of the fat image series was used to mitigate the difficulties of the dynamic contrast-enhancement when motion correcting myocardial perfusion MRI data. The proposed motion correction scheme provides a significant improvement compared with no motion correction and it performs similarly to the state-of-the-art methods^12^, as assessed by both the temporal smoothness of the myocardial time-intensity curves and the uniformity of the rest MBF parameter maps. The motion correction facilitates the computation of significantly more uniform perfusion maps after the correction of free-breathing image series, as expected for patients without perfusion defects under resting conditions. The benefit of the Dixon-based motion correction is that the fat images allow the initial estimation of the rigid body motion prior to the non-rigid correction. This is important as non-rigid corrections are computationally more expensive than rigid corrections and the initial rigid correction leads to a reduction in total registration time compared to the state-of-the art method. Increased sharpness in the tMIP images is also shown in Fig. 5 after both rigid and non-rigid Dixon MoCo as compared with the rigid only correction. This indicates that a rigid-only motion correction scheme is sub-optimal. A further benefit is that it allows direct registration to a template image rather than using an iterative approach such as the spatio-temporal denoising^12,31^.

The approach can also efficiently provide an initial estimate of rigid body motion, as it explicitly accounts for the dynamic contrast-enhancement by using the fat images. A number of other methods exist for the motion correction of myocardial perfusion data^12–16,26^. However, these either rely on assumptions on the data and pre-processing steps to account for the dynamic contrast-enhancement or do not explicitly account for it. The benefit of the proposed method is that the separation of the dynamic contrast-enhancement comes directly from the multi-echo Dixon reconstruction as the contrast agent does not affect the magnetisation of fat. This reduced reliance on data decomposition techniques or specific pre-processing steps is likely to mean that the proposed method is also more robust to the variations in different centres or acquisition parameters. Interestingly, the proposed motion correction approach benefits from larger amounts of fat, which is commonly encountered in patients with heart disease where obesity is often a comorbidity. These patients are typically more challenging to scan using conventional perfusion techniques and are a patient cohort for which the proposed mDixon approach may prove particularly beneficial compared to conventional perfusion acquisition techniques.

Although we have demonstrated good performance with this technique to estimate rigid body motion from the fat images, due to the sparse and unpredictable fat signal surrounding the heart, it may be difficult to achieve robust non-linear motion estimation using the fat images. To this end, in the second step we use the already rigid motion corrected water image series to estimate the non-rigid motion. To avoid registering images of vastly different contrast, each image from the water image series is registered to the image of corresponding contrast from a synthetic motionless image series created using a PCA decomposition^26,27^. This stage would not be possible without the earlier rigid correction as with a free-breathing acquisition the persistent motion would appear in these early principal components.

Furthermore, the three echoes were used to correct for the attenuation of the main bolus of the arterial input function caused by the *T*_2_* effects. Even though an increase in AIF peak signal intensity was observed after the T2* correction, this does not impact the quantitative MBF values obtained in this study as a dual-bolus acquisition is used. Although correction of both T1 saturation and T2*-related signal losses of the AIF are required for accurate perfusion quantification, the multi-echo design of the mDixon pulse sequence facilitates T2* correction directly from the imaging data. In the future mDixon could be combined with a dual sequence acquisition approach^20^ to allow single bolus acquisitions and in this case the T2* correction of the AIF would be relevant.

A further benefit of using Dixon-water fat separation for myocardial perfusion is that the epicardial fat signal is eliminated from the diagnostic water images. The effect that the epicardial fat signal can have on quantitative MBF maps is also demonstrated. Conventional fat suppression can be performed using fat suppression pre-pulses. However, this increases the specific absorption rate, is susceptible to B1 inhomogenities, and is more challenging to achieve with a linear profile order where the centre of k-space is acquired further away in time from the fat suppression pulse.

### Limitations

There is a lack of a consensus methodology for the evaluation of motion correction schemes as it is not feasible to have a ground-truth to compare with. In this work, the endpoint of the quantitative perfusion map was used to assess the success of the motion correction. There is however no ground truth values and it is difficult to compare with the existing literature on this topic. The imaging in this study is in 2D and as such it is not possible to account retrospectively for any through plane motion, this may influence the quantitative MBF values reported.

This feasibility study was performed in patients without ischaemic heart disease, at rest. The acquisition of three echoes leads to an increase in acquisition time. In order to still acquire the three requisite slices within one RR interval, a partial Fourier (factor = 0.63) reconstruction was employed. Dixon water-fat separation may be achieved using two echoes and other accelerations techniques, such as compressed sensing. Although this would reduce signal-to-noise ratio, it would also reduce the acquisition time, and will be investigated in further studies. Shortening the acquisition time will be important to enable the proposed approach in patients undergoing stress perfusion and will be the focus of future work. This will also allow a follow-up study in patients with suspected coronary artery disease.

## Conclusion

We have proposed a method which allows motion correction of free-breathing image series. The motion correction proved to be fast and robust as it negates the difficulties of the dynamic contrast-enhancement. It was shown that it is feasible to quantify the free-breathing image series in a reproducible manner after motion correction. Free-breathing acquisitions make the acquisition easier for both the scanner operator and the patient and have the potential to aid the clinical integration of quantitative perfusion analysis. The motion correction has the potential to be used as part of a fully-automated pipeline and is robust so it could be performed inline on the scanner. The echo images can be further used to estimate T2* related signal loss and the water-only images may be of higher diagnostic quality in patients with significant amounts of fat.

## Supplementary information

Supplementary Legends.

Supplementary Figure S1.

Supplementary Video S1.

Supplementary Video S2.

Supplementary Video S3.

Supplementary Video S4.

Supplementary Video S5.

Supplementary Video S6.

Supplementary Video S7.

Supplementary Video S8.

Supplementary Video S9.

Supplementary Video S10.

Supplementary Video S11.

Supplementary Video S12.

Supplementary Video S13.

Supplementary Video S14.

Supplementary Video S15.

## Data Availability

The datasets generated during and/or analysed during the current study are available from the corresponding author on reasonable request.
