# Supplementary material for: Feasibility of free-breathing quantitative myocardial perfusion using multi-echo Dixon magnetic resonance imaging
Source: Sci Rep. 2020 Jul 29;10:12684. doi: 10.1038/s41598-020-69747-9 (PMC7392760; doi:10.1038/s41598-020-69747-9)
Supplement: Supplementary file 1 — Supplementary Legends. [file 41598_2020_69747_MOESM1_ESM.pdf]

Feasibility of free-breathing quantitative myocardial perfusion using multi-echo Dixon magnetic resonance imaging

Cian M. Scannell (1), Teresa Correia (1), Adriana D. M. Villa (1), Torben Schneider (2), Jack Lee (1), Marcel Breeuwer (3) (4), Amedeo Chiribiri (1), Markus Henningsson* (1) (5)

Supplementary Figures

Figure S1. Example MBF maps computed with no motion correction, after motion correction using spatio-temporal denoising, and after using the proposed Dixon-based motion correction. These were chosen to show a range of performances of the different approaches.

Supplementary Videos

Video S1. The basal slice water image series before motion correction for patient A.

Video S2. The basal slice fat image series before motion correction for patient A.

Video S3. The basal slice water image series before motion correction for patient B.

Video S4. The basal slice fat image series before motion correction for patient B.

Video S5. The basal slice water image series after rigid motion correction for patient B.

Video S6. The basal slice fat image series after rigid motion correction for patient B.

Video S7. The basal slice water image series after both rigid and non-rigid motion correction for patient B.

Video S8. The basal slice water image series before motion correction for patient C.

Video S9. The basal slice water image series after proposed motion correction scheme for patient C.

Video S10. The mid-slice water image series before motion correction for patient D.

Video S11. The mid-slice water image series after proposed motion correction scheme for patient D.

Video S12. The apical slice water image series before motion correction for patient E.

Video S13. The apical slice water image series after proposed motion correction scheme for patient E.
