# Supplementary figures and images for: Feasibility of free-breathing quantitative myocardial perfusion using multi-echo Dixon magnetic resonance imaging
Source: Sci Rep. 2020 Jul 29;10:12684. doi: 10.1038/s41598-020-69747-9 (PMC7392760; doi:10.1038/s41598-020-69747-9)

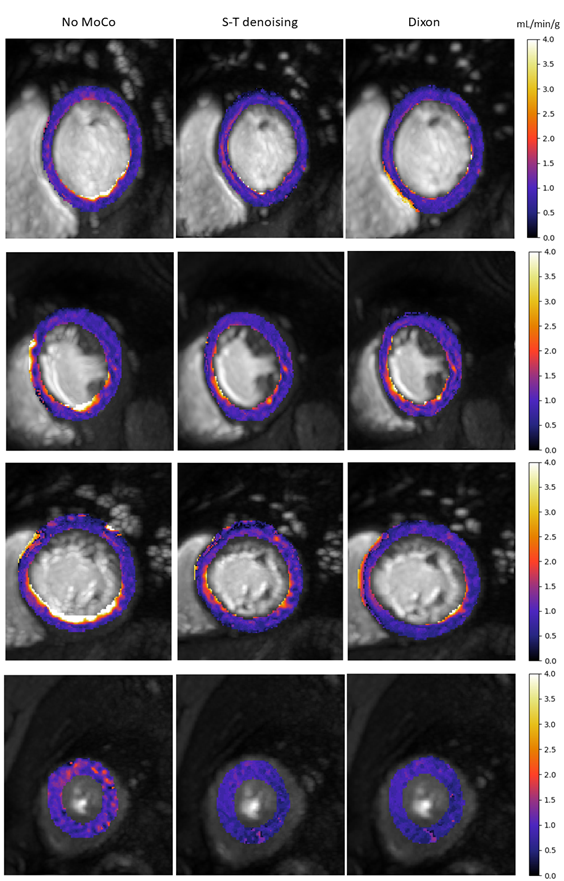

Supplement: Supplementary file 2 — Supplementary Figure S1. [file 41598_2020_69747_MOESM2_ESM.tiff]
